# Efficacy, safety and impact on antimicrobial resistance of duration and dose of amoxicillin treatment for young children with Community-Acquired Pneumonia: a protocol for a randomIsed controlled Trial (CAP-IT)

**DOI:** 10.1136/bmjopen-2019-029875

**Published:** 2019-05-22

**Authors:** Mark D Lyttle, Julia A Bielicki, Sam Barratt, David Dunn, Adam Finn, Lynda Harper, Pauline Jackson, Colin V E Powell, Damian Roland, Wolfgang Stohr, Kate Sturgeon, Mandy Wan, Paul Little, Saul N Faust, Julie Robotham, Alastair D Hay, Diana M Gibb, Mike Sharland

**Affiliations:** 1 Emergency Department, Bristol Royal Hospital for Children, Bristol, UK; 2 Faculty of Health and Applied Science, University of the West of England, Bristol, UK; 3 Paediatric Infectious Diseases Research Group, MRC Clinical Trial Unit at UCL, Institute for Infection and Immunity, St George’s University of London, London, UK; 4 MRC Clinical Trials Unit at UCL, London, UK; 5 Bristol Children’s Vaccine Centre, Schools of Population Sciences and Cellular and Molecular Medicine, University of Bristol, Bristol, UK; 6 Paediatric Emergency Medicine Department, Sidra Medicine, Doha, Qatar; 7 School of Medicine, Cardiff University, Cardiff, UK; 8 Emergency Department, Paediatric Emergency Medicine Leicester Academic (PEMLA) Group, Leicester, UK; 9 SAPPHIRE group, University of Leicester Department of Health Sciences, Leicester, UK; 10 NIHR CRN: Children, Guy’s and St Thomas' NHS Foundation Trust, London, UK; 11 Faculty of Medicine, University of Southampton, Southampton, UK; 12 NIHR Southampton Clinical Research Facility and NIHR Southampton Biomedical Research Centre, University Hospital Southampton NHS Foundation Trust, Southampton, UK; 13 HCAI and AMR Division, National Infection Service, Public Health England, London, UK; 14 Centre for Academic Primary Care, University of Bristol, Bristol, UK

**Keywords:** community-acquired pneumonia, antimicrobial resistance

## Abstract

**Introduction:**

Community-acquired pneumonia (CAP) is a common indication for antibiotic treatment in young children. Data are limited regarding the ideal dose and duration of amoxicillin, leading to practice variation which may impact on treatment failure and antimicrobial resistance (AMR). Community-Acquired Pneumonia: a randomIsed controlled Trial (CAP-IT) aims to determine the optimal amoxicillin treatment strategies for CAP in young children in relation to efficacy and AMR.

**Methods and analysis:**

The CAP-IT trial is a multicentre, randomised, double-blind, placebo-controlled 2×2 factorial non-inferiority trial of amoxicillin dose and duration. Children are enrolled in paediatric emergency and inpatient environments, and randomised to receive amoxicillin 70–90 or 35–50 mg/kg/day for 3 or 7 days following hospital discharge. The primary outcome is systemic antibacterial treatment for respiratory tract infection (including CAP) other than trial medication up to 4 weeks after randomisation. Secondary outcomes include adverse events, severity and duration of parent-reported CAP symptoms, adherence and antibiotic resistance. The primary analysis will be by intention to treat. Assuming a 15% primary outcome event rate, 8% non-inferiority margin assessed against an upper one-sided 95% CI, 90% power and 15% loss to follow-up, 800 children will be enrolled to demonstrate non-inferiority for the primary outcome for each of duration and dose.

**Ethics and dissemination:**

The CAP-IT trial and relevant materials were approved by the National Research Ethics Service (reference: 16/LO/0831; 30 June 2016). The CAP-IT trial results will be published in peer-reviewed journals, and in a report published by the National Institute for Health Research Health Technology Assessment programme. Oral and poster presentations will be given to national and international conferences, and participating families will be notified of the results if they so wish. Key messages will be constructed in partnership with families, and social media will be used in their dissemination.

**Trial registration number:**

ISRCTN76888927, EudraCT2016-000809-36.

**Strengths and limitations of this study**

- This well-powered multicentre trial will provide new data on efficacy of amoxicillin treatment strategies for uncomplicated community-acquired pneumonia in young children in developed settings in the postpneumococcal vaccine era.
- The Community-Acquired Pneumonia: a randomIsed controlled Trial (CAP-IT) is one of few randomised controlled trials to measure the impact of differing antibiotic treatment regimens on antimicrobial resistance.
- The pragmatic approach to eligibility employed by the CAP-IT trial is aligned with clinical practice and so will facilitate rapid knowledge translation.
- Although determining the optimal endpoint for pragmatic trials is challenging, use of an Endpoint Review Committee in the CAP-IT trial will strengthen the confidence in the results.
- The generalisability of the CAP-IT trial findings will be maximised by the diversity of the enrolled population, in both severity and setting.

## Introduction

Acute respiratory infections (including community-acquired pneumonia; CAP) are the most common indication for childhood antibiotic use in primary care and emergency departments (ED).^1–3^ Up to 40% of preschool children attend primary care for acute respiratory symptoms, with antibiotics prescribed in 30%.^3 4^ One-third of childhood ED visits are for acute respiratory symptoms or fever; up to 15% are diagnosed with CAP, with highest antibiotic prescription rates in those aged <5 years.^5–7^ ED attendances and hospital admissions for respiratory illness in young children increase annually, and two-thirds of antibiotics for hospitalised children aged 1–5 years are for CAP.^5 8 9^ The healthcare cost of childhood CAP in England is estimated at £8 million/year, though total annual societal costs are up to £17 million.^10 11^

CAP is a differential diagnosis in any child presenting with fever, respiratory symptoms and focal chest signs. No gold standard laboratory, microbiological or radiological tests distinguish bacterial from viral infection; diagnosis and treatment decisions are based on clinical criteria.^12–14^ *Streptococcus pneumoniae* is the most commonly implicated bacterial pathogen, even in settings with routine pneumococcal vaccination (PCV).^15–17^ Children have high rates of bacterial colonisation and carriage of resistant organisms which may be transmitted, with particular risks for vulnerable individuals.^18–21^ Interventions to maintain low antimicrobial resistance (AMR) levels in children may therefore have wider benefits.

Amoxicillin is the recommended antibiotic for childhood CAP in several international guidelines^12 22–24^; however, there are insufficient data to inform dose, duration and impact on AMR. The British National Formulary for Children recommends amoxicillin thrice daily at 40–80 mg/kg/day. Twice daily dosing is widely recommended due to improved adherence and non-inferiority, including by the WHO.^22–26^ Shorter treatment courses are effective in trials performed in low/middle-income country settings, though diagnostic and eligibility criteria limit generalisability of findings.^27 28^ Trials from developed settings predate PCV or include populations with high penicillin resistance rates. There are therefore no robust data to inform treatment duration, leading to variation between 3 and 7 days.^23 24 27–30^ Assessment of treatment efficacy is challenging, as only half of patients recover after 10 days, and 90% take up to 4 weeks to recover fully.^4 31 32^ While ongoing ‘minimal’ symptoms may trigger retreatment (deemed treatment failure) relatively frequently, the most widely used efficacy measure is re-exposure to antibiotics for up to 4 weeks, with recent reported rates of 15%.^29 31 33^

The impact of antibiotics on colonisation with resistant bacteria is complex and dynamic.^34–37^ Insufficiently high dosing could promote selection of resistant pathogens, and while the greatest effect on bacterial load is achieved early, resistant isolates emerge after 4–5 days.^38 39^ Combined effectiveness and resistance data related to antibiotic dose and duration would inform antimicrobial stewardship and childhood CAP treatment strategies.

The Community-Acquired Pneumonia: a randomIsed controlled Trial (CAP-IT) will evaluate efficacy, safety and effect on AMR of different durations and doses of oral amoxicillin treatment for young children with uncomplicated CAP. The specific primary objectives are to determine whether (1) 3 days is non-inferior to 7 days, and (2) 35–50 mg/kg/day is non-inferior to 70–90 mg/kg/day.

## Methods

**Figure 1:**
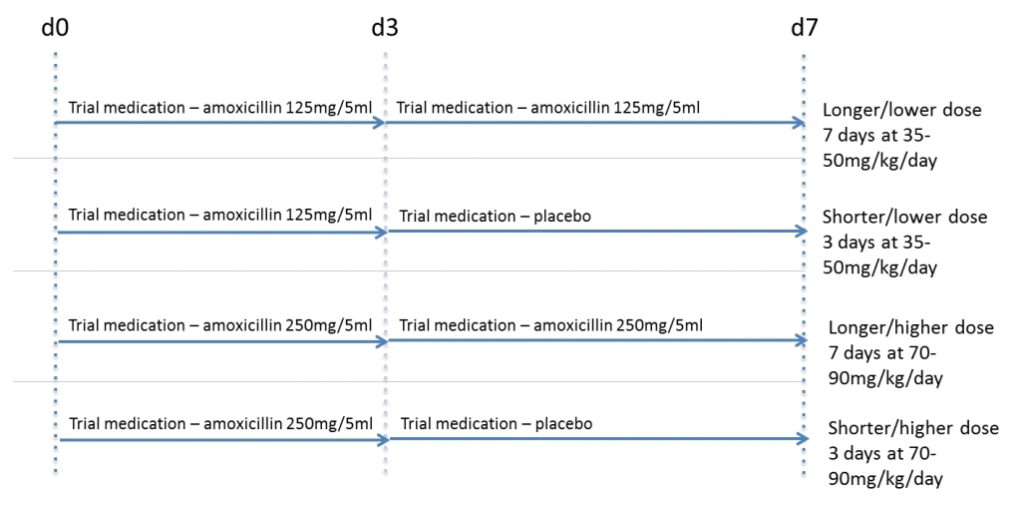
Possible treatment arms in the CAP-IT trial. CAP-IT, Community-Acquired Pneumonia: a randomIsed controlled Trial.

This multicentre, randomised, double-blind, placebo-controlled 2×2 factorial non-inferiority trial aims to enrol 800 patients over 2 years. Amoxicillin dose and duration are assigned simultaneously, resulting in four treatment arms (figure 1). This protocol has been written in accordance with the Standard Protocol Items: Recommendations for Interventional Trials checklist.^40^

### Patient and public involvement

Parents of young children have been involved throughout the development and delivery of the CAP-IT trial. In developing the research question, they advised that shorter antibiotic courses would be welcomed if equally effective, due to difficulties in giving medicine (due to palatability, or challenges with day care and daytime doses). For the same reasons, parents supported twice daily dosing. A key need identified during trial development was support for families, including clear symptomatic safety netting, close contact and availability of a contact number for the local research and clinical teams. Parents reviewed and provided input on all information materials to ensure they were clear and easy to understand. The CAP-IT Trial Steering Committee (TSC) has a parent member, who contributes to discussions as the trial progresses, and will advise on best methods of disseminating results to families as we approach the analysis phase.

### Trial setting

Sites throughout the UK and Ireland are participating from Paediatric Emergency Research in the UK and Ireland (PERUKI; www.peruki.org)^41^ and General and Adolescent Paediatric Research in the UK and Ireland (GAPRUKI; www.gapruki.org.uk). Participating sites are tertiary or secondary hospitals with EDs and inpatient areas where potentially eligible children are managed; participating centres are listed on the trial website (www.capitstudy.org.uk). Centres are selected based on research infrastructure, likely number of recruits, participation in feasibility work and proposed training strategies. Site principal investigators (PI) are qualified by education, training and experience to assume responsibility for proper conduct of the trial.

### Trial population

Participants may be enrolled from EDs, observation units, paediatric assessment units (PAU) or inpatient wards, provided they fulfil eligibility criteria.

#### Inclusion criteria

Children are eligible if they are aged ≥6 months, weigh 6–24 kg, have a clinical diagnosis of CAP (box 1) and will be treated with amoxicillin as the sole antibacterial agent on hospital discharge. Children must have received none or less than 48 hours of treatment with only beta-lactam antibiotics at enrolment.

#### Exclusion criteria

Children are ineligible if they have (1) severe underlying chronic disease (including sickle cell anaemia, immunodeficiency, chronic lung disease, cystic fibrosis), (2) penicillin allergy or other contraindication to amoxicillin, (3) complicated pneumonia (shock, hypotension, altered mental state, ventilatory support, empyema, pneumothorax, pulmonary abscess), or (4) bilateral wheezing without focal chest signs.

Box 1 **Definition of clinical diagnosis of CAP**

Clinical diagnosis of community-acquired pneumonia (CAP) is defined as all of the following:

1. Cough (reported by parents/guardians within 96 hours before presentation).
2. Temperature ≥38°C measured by any method or reported fever within 48 hours before presentation.
3. Signs of laboured/difficult breathing or focal chest signs (one or more of the following):

- Nasal flaring.
- Chest retractions.
- Abdominal breathing.
- Focal dullness to percussion.
- Focal reduced breath sounds.
- Crackles with asymmetry.

### Outcome measures

The primary outcome is clinically indicated treatment with systemic antibiotic other than trial medication for respiratory tract infection (including CAP) up to 4 weeks after randomisation. Reason and clinical indication are adjudicated by an Endpoint Review Committee (ERC), comprising independent and non-independent members. Independent members, including the ERC chair, are paediatricians independent of the trial and not involved in the clinical care of participants. Non-independent members include the chief investigator, project lead and trial physician. ERC members review clinical narrative summaries of retreatment primary endpoint events, supportive data from patient trial diaries, unscheduled visit forms and serious adverse event (SAE) forms; they adjudicate whether additional antibiotics were prescribed due to CAP (early failure, relapse or recurrence), new bacterial infection (respiratory tract infection or other), intolerance of trial medication or another reason. Final adjudication is based on consensus of the independent ERC members.

Secondary outcomes include (1) severity and duration of parent-reported CAP symptoms, (2) specified amoxicillin-related adverse events (AE; thrush, skin rashes and diarrhoea), (3) phenotypic resistance to penicillin at 4 weeks in nasopharyngeal (NP) *S. pneumoniae* isolates, and (4) adherence to trial medication.

### Screening, randomisation, recruitment and consent

**Figure 2:**
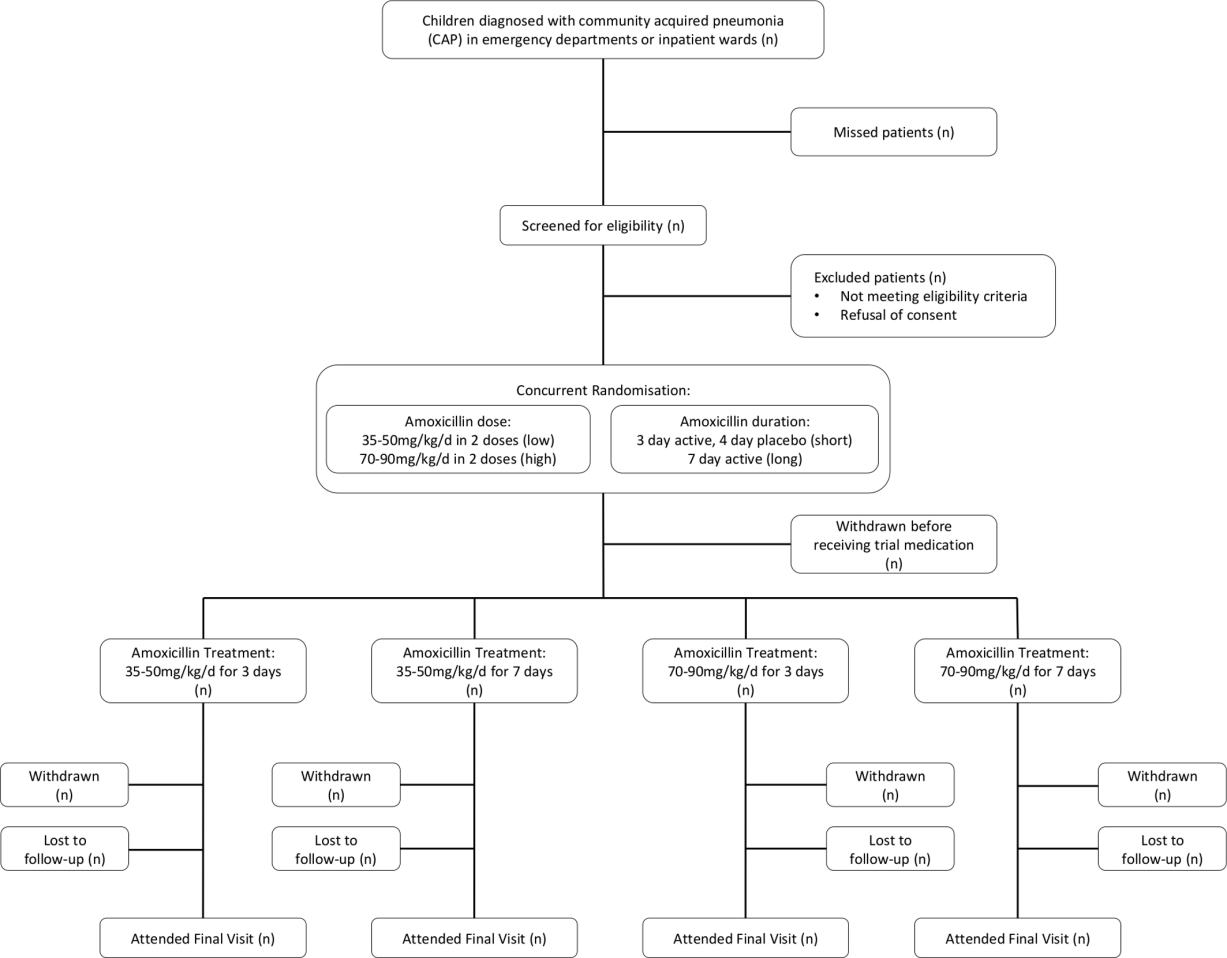
CAP-IT trial flow chart. CAP-IT, Community-Acquired Pneumonia: a randomIsed controlled Trial.

**Figure 3:**
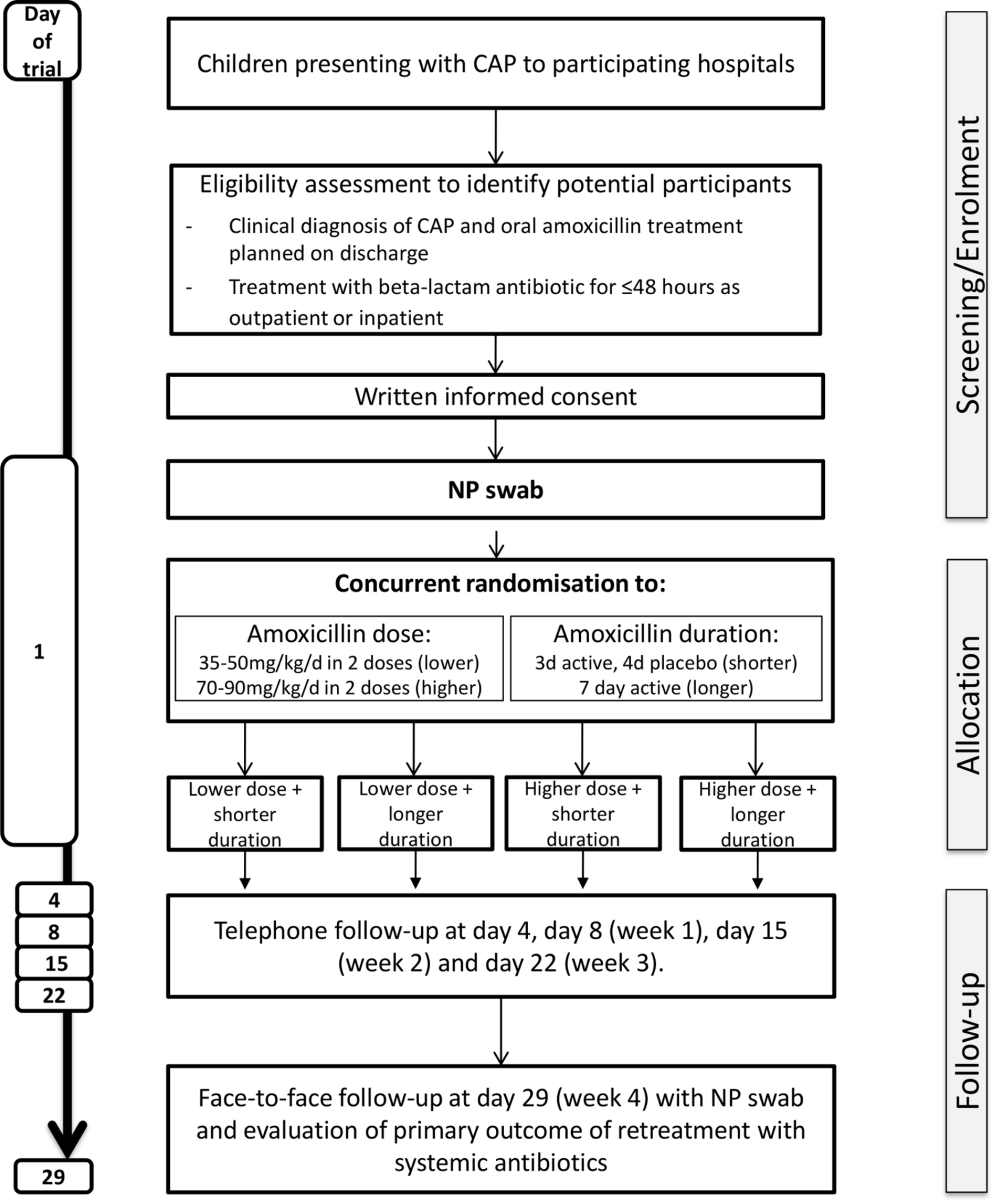
CAP-IT trial schema. CAP-IT, Community-Acquired Pneumonia: a randomIsed controlled Trial; NP, nasopharyngeal.

**Figure 4:**
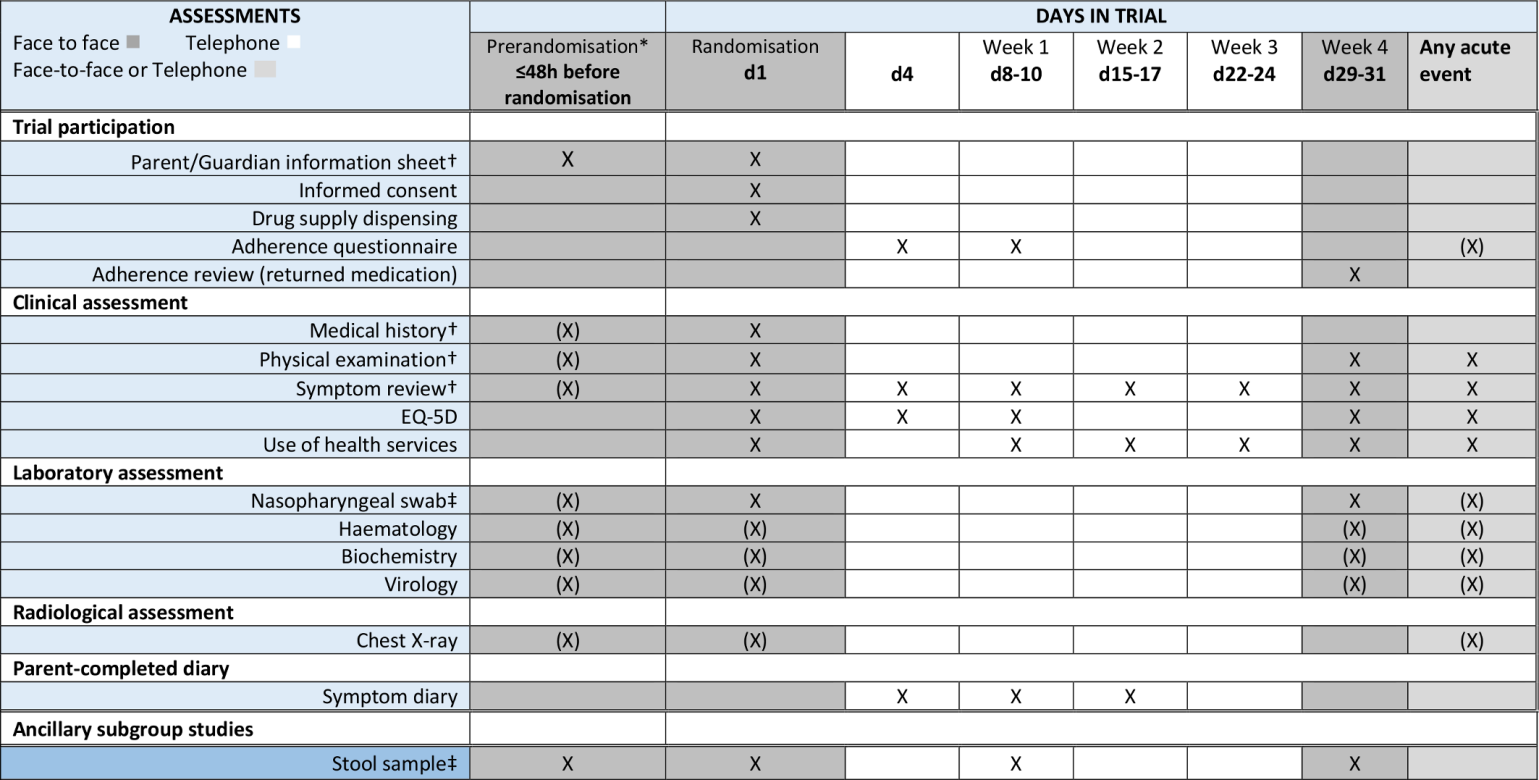
CAP-IT trial assessment schedule (X) indicates tests that may be done if the child’s condition requires it or allows it, but are not mandatory. *Assessments in this column only undertaken for potential participants receiving inpatient antibiotic treatment. †May be done any time before enrolment discussion. ‡Taken before starting antibiotics where possible. CAP-IT, Community-Acquired Pneumonia: a randomIsed controlled Trial.

The trial flow chart is provided in figure 2. Potential participants may require hospital admission for initial CAP management or may be discharged immediately from an ED or PAU. As antibiotic treatment duration is an eligibility criterion, additional eligibility assessment procedures are undertaken for those receiving antibiotics while in hospital; these differences are outlined in the following sections, and in figures 3 and 4.

#### Screening

Potential participants are identified and screened during initial clinical assessment. Participants discharged directly from an ED or PAU undergo all trial procedures prior to discharge. Children given antibiotics while in hospital have a secondary screening episode. Participating centres keep anonymised logs of screened children, including those who were not approached and those who declined participation, which are used to identify any issues for the trial, or at a given site.

#### Consent

Extensive information is available for recruiting sites, including printed and video materials (accessible at www.capitstudy.org.uk); all materials have NHS Research Ethics Committee (REC) approval. Written informed consent is obtained by clinical or research staff from a parent/guardian after explanation of the aims, methods, benefits and potential hazards of the trial. Families may decline participation in all or any aspects of the trial, at any time and for any reason, without incurring any penalty or affecting treatment. Signed consent forms are kept by the site and a copy given to the family. A letter is sent to the general practitioner (GP) informing them of the trial and the child’s involvement.

#### Enrolment process

At enrolment, recruiting staff review trial materials with parents/guardians; these include a symptom diary, participant information sheet (PIS), investigational medicinal product (IMP) administration instructions and contact details for the trial team. The symptom diary collects information on clinical and non-clinical elements including cough, breathing, temperature, AEs, IMP administration, additional antibiotics, any other medication, time off work/childcare and other health service use. Relevant elements are incorporated into baseline data collected by recruiting staff on enrolment clinical report forms. This is done with parents/guardians to facilitate their understanding of diary content; other baseline data include demographics, symptoms, underlying disease, antibiotic exposure in the preceding 3 months, chest findings and quality of life measures. Participants are registered on the online trial database; database access is controlled through authorised usernames and passwords and all patient identifiable details are confidential.

An NP swab is collected at enrolment. *S. pneumoniae* is identified using culture-based techniques, AMR is detected using minimal inhibitory concentration-based techniques and respiratory viruses are detected using molecular techniques. Stool samples are collected in sites participating in ancillary studies, provided specific consent is given. There are no other mandatory radiological or laboratory tests; if done as part of routine clinical care, results of haematological, biochemical and chest radiograph investigations are recorded.

#### Randomisation

Participants are allocated 1:1 to each of the two factorial randomisations, stratified according to whether or not they receive any non-trial antibiotics in hospital before being enrolled. A randomisation list is computer generated based on random permuted blocks by the trial statistician. Based on this list, trial medication (including placebo) is labelled and packaged in a blinded manner by an independent supplier and delivered to trial sites; treatments are randomly assigned by taking the next sequentially numbered treatment from the relevant supply. As it is difficult to taste-match suspensions of amoxicillin and placebo, one brand of amoxicillin is used for all participants for the first 3 days, followed by a second bottle for days 4–7 containing either a second brand of amoxicillin or placebo; both form a yellow-coloured similar tasting suspension, and parents are instructed to expect some change in taste after the first 3 days.

### Trial treatments

Trial treatment starts on the day of enrolment, with the first dose given before discharge where possible. Children receive 3 or 7 days of amoxicillin, and suspension is given twice daily with dosing by weight band; doses are 35–50 or 70–90 mg/kg/day. To ensure blinding, amoxicillin suspensions of different strengths (125 or 250 mg/5 mL) are used so that dose volume is the same regardless of treatment arm.

IMP is stored separately from routine medicines, and is dispensed in a box containing one bottle of amoxicillin and two bottles of either amoxicillin or placebo (blinded to contents). Colour labels indicate which should be used on days 1–3 and days 4–7. The importance of adherence is reinforced when dispensing IMP and during follow-up contacts. Adherence is assessed using the symptom diary, at telephone follow-up contacts, and at final follow-up when families return unused IMP.

IMP may be stopped early for reasons including toxicity, clinical change requiring treatment modification, or withdrawal of consent. All concomitant medications are allowed; if an essential medication interacts with amoxicillin, the IMP is stopped. Although participants are not required to give a reason for discontinuing IMP, a reasonable effort is made to establish this reason.

Situations necessitating unblinding are likely to be rare. Severe allergic reactions occur early during amoxicillin exposure; immediate discontinuation and trigger avoidance is recommended. Delayed reactions are generally mild and resolve with discontinuation. Where retreatment is necessary, unblinding is unlikely to impact on antibiotic choice and is therefore unnecessary. If necessary, unblinding is done using an online emergency unblinding form.

### Follow-up

The trial assessment and follow-up schedule is shown in figures 3 and 4. Telephone contact occurs on days 4, 8–10, 15–17 and 22–24; a face-to-face visit occurs within 2 days of day 29. At each contact clinical signs and symptoms are reviewed, as are adverse treatment effects, acute illnesses requiring medical assessment, new antibiotics and IMP adherence. At each face-to-face visit an NP swab is collected; if there are any CAP symptoms, examination findings and physiological parameters are recorded. If a face-to-face visit is not possible for final follow-up, it is attempted by telephone. If it is not possible to contact families despite reasonable efforts, relevant data are sought from the GP if consent has been given to do so.

Additional healthcare contacts may be necessary. Parents/guardians liaise with the site trial team if they are considering acute clinical assessment during the follow-up period, though they are advised to seek immediate emergency assessment if they feel this is required. Clinician judgement determines whether investigation, treatment or hospitalisation is required. Following any unscheduled assessment, symptoms, health service utilisation and IMP adherence are reviewed by telephone, and face-to-face visits arranged if necessary.

Parents/guardians who discontinue IMP are encouraged to adhere to the follow-up schedule, and the Sponsor is informed. If follow-up is stopped early, data already collected are kept for analysis, and the children are not replaced in the trial. Samples already obtained are processed unless parents/guardians request otherwise.

### Sample size and power

The sample size is based on demonstrating non-inferiority for the primary endpoint for each duration and dose. Although inflation factors have been advocated for factorial trials to account for interaction between the interventions or a reduction in the number of events, this is not necessary if either randomised intervention has a null effect (the underlying hypothesis with a non-inferiority design), as marginal analyses can then be conducted. Assuming a 15% primary outcome event rate, 8% non-inferiority margin assessed against an upper one-sided 95% CI, 90% power and 15% loss to follow-up, 800 children will be randomised. This is considered a minimum sample size; if resources permit, recruitment may continue above this number to increase statistical power and precision, with the approval of the TSC.

### Analysis plan

The primary analysis will be by modified intention to treat (ITT), including all participants who take at least one IMP dose. The primary endpoint will be analysed using time-to-event methods, controlling for previous antibiotic exposure. Potential interaction effects between dose, duration and previous antibiotic exposure will be examined. For some secondary outcomes, including AEs and resistance, on-treatment analyses will be performed as well as ITT analyses.

The primary analysis of the primary endpoint will include only clinically indicated additional systemic antibiotic for respiratory tract infection (including CAP) as adjudicated by the ERC. Sensitivity analyses will be performed to include (1) all systemic antibacterial treatments other than IMP regardless of reason and indication (to guard against bias), and (2) only ERC-adjudicated clinically indicated systemic antibacterial treatment prescribed specifically for CAP (to provide clinical reassurance).

A subgroup analysis will consider CAP severity at presentation, and repeat the main efficacy analysis limited to participants with more severe disease. This will provide reassurance that an overall null effect (if observed) is not due to a dilution effect arising from inclusion of children with mild disease of viral aetiology, an important consideration as CAP is diagnosed clinically.

Lower dose and shorter duration will be considered ‘non-inferior’ to higher dose and longer duration, respectively, if the upper limit of the one-sided 95% CI for the difference in the proportion of children with the primary endpoint at day 29 is less than the non-inferiority margin of 8%. However, inference will be based primarily on point estimates and CIs rather than binary classification of a ‘non-inferior’ or ‘not non-inferior’ outcome.^42^

### Ancillary studies

#### Impact on gastrointestinal microflora

Stool samples will be collected at enrolment and final follow-up from 200 children to evaluate the impact of amoxicillin exposure on different microbial communities, including AMR in gastrointestinal commensal flora. The baseline sample is collected prior to or as soon as possible after starting amoxicillin.

#### Symptom diary completion methodology

While widespread access to the internet and mobile devices suggest web-based questionnaires may be acceptable and reliable, data supporting this are lacking. As it is unclear which is best in terms of completion rates we will quasirandomise participants to electronic or paper questionnaires based on gender and site. We will compare overall data completeness, and completeness of individual items between paper and electronic diaries. Data supporting use of one or other approach will be important for many trials eliciting primary or key secondary parent-reported outcomes through the use of symptom diaries.

### Safety reporting

Definitions for AEs and adverse reactions (AR) of the European Union (EU) Directive 2001/20/EC Article 2 based on the principles of Good Clinical Practice (GCP) apply to this trial. The symptom diary prompts for known amoxicillin ARs including gastrointestinal symptoms and rash; AEs that lead to cessation of trial treatment are also reported. When an AE or AR occurs, the PI assesses whether this is an SAE using supplied definitions, and the level of severity using provided toxicity grading. Where an SAE has occurred, an SAE form is completed and the Sponsor notified within 24 hours of the PI becoming aware. Children are followed up after an SAE until complete clinical recovery or until the event has stabilised. Staff follow their institution’s procedure for local notification requirements. PIs assess causality of SAEs in relation to IMP; assessment of expectedness is completed based on common toxicities listed in the Summary of Product Characteristics; if a serious AR (SAR) is unexpected, the event is classified as a suspected unexpected SAR (SUSAR) and reported to the Medicines and Healthcare products Regulatory Agency (MHRA), REC and site PIs. Medically qualified staff at the Sponsor and the CI (or medically qualified delegate) review all SAE reports. In case of disagreement with regard to causality, both opinions are provided in any reports. All SAEs are reported to the MHRA and REC in the annual Development Safety Update Report.

### Trial monitoring

Quality assurance and quality control are based on a formal risk assessment, to ensure all elements are performed in line with applicable regulatory requirements. Site initiation visits are done to deliver trial-specific training to key clinical and research personnel, which is cascaded to all relevant staff; training and delegation logs are monitored for completeness. Screening, randomisation and consent rates are monitored by the trial team at the Medical Research Council Clinical Trials Unit (MRC CTU) at University College London (UCL), and data are checked for consistency, missing data points or possible errors, with suspect data returned as queries to ensure reliability and validity. PIs allow trial-related monitoring including audits, ethics committee review and regulatory inspections, by providing direct access to source data and documents as required. The principles of the UK Data Protection Act (DPA) are followed.

The Trial Management Group (TMG) comprises the CI, clinical and non-clinical investigators, and the CAP-IT MRC CTU at UCL team. The TMG is responsible for the day-to-day running and management of the trial. The TSC has membership from the TMG plus independent members, including an independent chair, and provides overall guidance and advice. The Independent Data Monitoring Committee (IDMC) is the only group which sees confidential accumulating data for the trial by randomised group. Formal stopping rules are not used although the IDMC Charter specifies guidelines for when to alert the TSC to consider modifying the trial design. These guidelines are conservative to guard against premature changes to trial design from early inspection of the data.

### Regulatory compliance

This trial is conducted in compliance with GCP principles as laid down by the Commission Directive 2005/28/EC, Statutory Instrument 2004 No 1031: Medicines for Human Use (Clinical Trials) Regulations 2004 as amended, the UK DPA (DPA number: Z5886415) and the NHS Research Governance Framework for Health and Social Care. Sites inform the Sponsor as soon as they are aware of any possible serious breach of compliance, so that the Sponsor can report this as per regulatory requirements. This is a Clinical Trial of an IMP as defined by EU Directive 2001/20/EC. The CTA number is 17141803 and the EudraCT number is 2016-000809-36.

## Ethics and dissemination

The protocol, PIS, consent form and all relevant documentation were approved centrally by the National Research Ethics Service, West London and GTAC REC (reference: 16/LO/0831; 30 June 2016). The results of the CAP-IT trial will be published in peer-reviewed journals read by health professionals managing childhood CAP in the UK and internationally, and in a report published by the National Institute for Health Research Health Technology Assessment programme. To maximise impact internationally, oral and poster presentations will be given to relevant national and international conferences. Once the trial is completed, all participating families will be notified of the results if they wish. The social media presence of the organisations involved, including PERUKI (@PERUKItweep), will be used to disseminate key messages.

## Discussion and trial status

The first participant was enrolled in February 2017; as of 31 January 2019, a total of 690 patients have been enrolled from 30 sites. Recruitment is scheduled to finish in April 2019, and analysis to be completed by August 2019. The trial will end after the last follow-up visit of the last randomised participant.

Three significant changes to the CAP-IT analysis plan have been made during the course of the trial (Protocol Version 4.0, 4 December 2018) based on blinded emerging trial data. First, although patients were originally intended to be stratified based on whether they received inpatient antibacterial treatment or were discharged directly from hospital, all patients will now be jointly analysed due to significant phenotypic overlap. Subgroup efficacy analyses will be performed to consider severity of CAP at presentation. Second, the primary endpoint definition has been made more specific from ‘all antibiotic prescriptions given during follow-up’ to ‘clinically indicated antibiotic prescriptions for respiratory tract infections (including CAP) as adjudicated by an ERC’. Finally, the non-inferiority margin was adjusted as the primary endpoint event rate had been substantially underestimated.

The CAP-IT trial will provide important and robust evidence for amoxicillin treatment strategies for childhood CAP in developed settings after PCV introduction. Its factorial nature will inform amoxicillin dose and duration, and the optimal regimen for minimal AMR selection. Combined with the results of ongoing trials (including SAFER^43^ and SCOUT-CAP [accessible at https://clinicaltrials.gov/ct2/show/NCT02891915]) in similar healthcare systems, findings from the CAP-IT trial will improve and streamline cost-effective care on a global scale.^24^

The CAP-IT trial provides an important opportunity for learning and infrastructure development in acute paediatric trials in the UK and Ireland. Recruiting patients to a placebo-controlled randomised trial in EDs, PAUs, observation units and inpatient wards has required close working across clinical (emergency medicine and general paediatric) and research teams, and research networks. To date, successful recruitment has been led through close communication between the CTU and participating sites, with leadership from PERUKI, providing frequent opportunities to share best practice in overcoming any obstacles to trial delivery. This includes regular contact with sites, and a metaplanning exercise prior to the second winter of recruitment. Key obstacles identified included research training (GCP and study specific), out-of-hours recruitment, availability of site research staff and delays to discharge caused by study processes. To overcome these, a number of new initiatives were developed, including streamlining of enrolment processes, to enable families to have the opportunity to participate in this important trial.

## Supplementary Material

Reviewer comments
