# Supplementary material for: Efficacy, safety and impact on antimicrobial resistance of duration and dose of amoxicillin treatment for young children with Community-Acquired Pneumonia: a protocol for a randomIsed controlled Trial (CAP-IT)
Source: BMJ Open. 2019 May 22;9(5):e029875. doi: 10.1136/bmjopen-2019-029875 (PMC6538022; doi:10.1136/bmjopen-2019-029875)
Supplement: Reviewer comments [file bmjopen-2019-029875.reviewer_comments.pdf]

PEER REVIEW HISTORY

BMJ Open publishes all reviews undertaken for accepted manuscripts. Reviewers are asked to complete a checklist review form (**http://bmjopen.bmj.com/site/about/resources/checklist.pdf**) and are provided with free text boxes to elaborate on their assessment. These free text comments are reproduced below.

ARTICLE DETAILS

**TITLE (PROVISIONAL)**	Efficacy, safety, and impact on antimicrobial resistance of duration and dose of amoxicillin treatment for young children with Community-Acquired Pneumonia: a protocol for a randomIsed controlled trial (CAP-IT)
**AUTHORS**	Lyttle, Mark; Bielicki, Julia; Barratt, Sam; Dunn, David; Finn, Adam; Harper, Lynda; Jackson, Pauline; Powell, Colin; Roland, Damian; Stohr, Wolfgang; Sturgeon, Kate; Wan, Mandy; Little, Paul; Faust, Saul; Robotham, Julie; Hay, Alastair; Gibb, Diana; Sharland, Mike

This article was not externally reviewed at BMJ Open. Protocols that have been independently assessed prior to submission to BMJ Open are usually fast-tracked to publication on the grounds that further substantial changes will not be possible. This independent assessment will usually be external, independent review for both a substantial grant award from a non-commercial or government funder and ethics approval.
